# Tiny caterpillars assess threats by the footsteps of their enemies

**DOI:** 10.1242/jeb.252329

**Published:** 2026-06-04

**Authors:** Emilie Mauduit, Sarah M. Matheson, Jayne E. Yack

**Affiliations:** Department of Biology, Carleton University, 1125 Colonel By Drive, Ottawa, ON, Canada, K1S 5B6

**Keywords:** Vibration, Vibroscape, Predator–prey interaction, Discrimination, Territorial, Defensive behaviour

## Abstract

Insects inhabit complex vibroscapes shaped by substrate-borne vibrations from multiple biotic and abiotic sources. One underappreciated topic is how vibrations function in predator–prey interactions. Tiny warty birch caterpillars (*Falcaria bilineata*) are known to produce complex vibratory signals to defend leaf-tip territories against conspecifics, raising the question of whether vibratory signalling and sensing also play roles in predator–prey interactions. We staged encounters between resident neonate caterpillars and three natural intruders: conspecifics, ladybird beetle larvae and adult ladybird beetles, while simultaneously recording behaviour and substrate-borne vibrations. Resident caterpillars showed three key responses – vibratory signalling, freezing and dropping – but these responses varied strongly with intruder identity and stage of encounter. Residents signalled vigorously toward conspecifics, with rates escalating as intruders approached their territories. In contrast, encounters with predators evoked predator-specific defensive strategies including freezing and dropping. Adult ladybird beetles, which caused high mortality (43%), elicited rapid escape responses and subsequent territory abandonment, while ladybird beetle larvae, which caused no mortality, triggered slower responses with initial signalling that ceased upon closer approach. Critically, vibrations generated by the ‘footsteps’ of each approaching intruder type produced distinct vibratory ‘signatures’, differing in amplitude, spectral and temporal characteristics. Resident caterpillars also initiated defensive responses before physical contact, often when intruders were still centimetres away. Together, these findings demonstrate that these miniature larvae, no larger than ∼1–2 mm, thrive in complex vibroscapes where vibrations not only function to advertise territory ownership against conspecifics but also provide essential early-warning cues enabling sophisticated threat assessment and context-appropriate defensive responses in predator-rich environments.

## Teaser

**Highlighted Article:** Tiny neonate warty birch caterpillars use leaf vibrations to identify enemies and deploy threat-appropriate defensive strategies.

## INTRODUCTION

Insects live in a vibratory world, where substrate-borne vibrations are a key component of their sensory ecology. Historically considered a private and specialized communication channel, vibratory sensing and communication is now recognized as widespread and ecologically relevant (Cocroft and Hamel, 2010; Cocroft and Rodríguez, 2005; Hill, 2001; Turchen et al., 2022; Virant-Doberlet et al., 2014, 2019, 2023). Insects and other invertebrates are surrounded by vibrations from numerous biotic (e.g. communication signals, incidental movements) and abiotic (e.g. wind, rain) sources (Barth, 1998; Virant-Doberlet et al., 2014; Yack, 2016), which together create a rich and dynamic vibratory landscape, known as the vibroscape (Šturm et al., 2019; Virant-Doberlet et al., 2023). These complex vibroscapes present challenges for insects, requiring them to filter out irrelevant noise while accurately detecting crucial signals and cues from a noisy background (Virant-Doberlet et al., 2023; Yack, 2016). The growing field of invertebrate vibratory sensory ecology explores how they detect, process and respond to substrate-borne vibrations in their environment (Elias and Mason, 2014; Virant-Doberlet et al., 2023). One particularly important aspect of vibratory sensory ecology is its role in predator–prey interactions (Virant-Doberlet et al., 2019).

Predator–prey interactions drive evolutionary change, shaping both sensory systems and behavioural strategies across diverse taxa (Edmunds, 1974; Ruxton et al., 2018). While prey have evolved a wide array of anti-predator adaptations including crypsis, mimicry, aposematism or defensive signalling, predators have evolved counter-strategies such as search image formation or resistance to toxicity (Edmunds, 1974; Low et al., 2021; Ruxton et al., 2018). Sensory systems are fundamental to predator–prey dynamics (Sillar et al., 2016; Stevens, 2013) and although visual, chemical and auditory sensory modalities have been well documented in these dynamics (Sillar et al., 2016; Ruxton et al., 2018), predator–prey interactions in the vibroscape remain comparatively underexplored (Hill, 2008; Virant-Doberlet et al., 2019). There is increasing evidence for vibration-mediated predator–prey interactions in arthropods, including predators and parasitoids eavesdropping on prey (Brownell and van Hemmen, 2001; Devetak, 2014; Meyhöfer et al., 1994; Pfannenstiel et al., 1995), predators luring prey through vibratory mimicry (Wignall and Taylor, 2011), and prey avoiding enemy attacks through vibratory crypsis by staying motionless (Djemai et al., 2001; Kojima et al., 2012; Lohrey et al., 2009) or defensive signalling (Cocroft, 1996; DeVries, 1991; Hager and Kirchner, 2013; Hartbauer, 2010). However, such examples remain rare relative to the extensive literature on visual, chemical and acoustic predator–prey interactions. In particular, there is a need to incorporate more models under ecologically relevant contexts, and how prey recognize and discriminate between different types of intruders based on substrate-borne cues (Hill, 2008; Virant-Doberlet et al., 2019). In this study, we explored the importance of vibratory interactions between vibratory caterpillars and their natural predators.

Caterpillars represent a compelling model for studying vibratory predator–prey interactions. As major herbivores and a key prey group across ecosystems, they are targeted by a wide range of natural enemies, including invertebrate predators and parasitoids (e.g. wasps, flies, mantids, stink bugs, dragonflies, ants, spiders) and vertebrates (e.g. birds, bats, lizards, rodents, toads) (Greeney et al., 2012; Heinrich, 1993; Montllor and Bernays, 1993). In response to this high predation pressure, caterpillars have evolved an impressive arsenal of anti-predator adaptations, ranging from visual and chemical defences – such as cryptic coloration, mimicry, warning signals, urticating hairs and toxic secretions – to behavioural strategies such as thrashing, dropping and constructing silk shelters (Gentry and Dyer, 2002; Lederhouse, 1990; Marquis and Koptur, 2022). However, most studies of caterpillar defence have focused on visual and chemical strategies, while vibratory interactions remain underexplored. Yet caterpillars are substrate-bound organisms residing primarily on or within plants (e.g. leaves, twigs) or silk structures (e.g. silk mats on leaves, tents), and they have limited vision (England et al., 2026). This makes them especially reliant on substrate-borne vibrations to perceive and respond to environmental cues (Yack, 2022; Yack and Yadav, 2022). Research over the past decades has shown that caterpillars can both produce and detect substrate-borne vibrations in various contexts, including recruitment (Yadav et al., 2017), manipulating ants in parasitic or mutualistic interactions (DeVries, 1991; Travassos and Pierce, 2000), and territorial or spacing behaviour (Fletcher et al., 2006; Matheson et al., 2025; Scott et al., 2010; Yack et al., 2001, 2014). Some studies suggest that vibrations may play an important role in predator detection and defence among caterpillars. For example, some caterpillars may freeze upon being approached or actively produce vibratory signals when disturbed (Guedes et al., 2012; Low, 2008). Despite increasing interest in vibration-based predator–prey interactions in larval insects, many aspects remain poorly understood, including whether they can detect and distinguish between vibration sources, and whether they use signalling in a defensive context. In this study, we addressed these questions in neonates of the warty birch caterpillar, *Falcaria bilineata* (Lepidoptera: Drepanidae).

The warty birch caterpillar is a Nearctic species that feeds on the leaves of birch (*Betula* spp.) and alder (*Alnus* spp.) (Betulaceae) (Rose and Lindquist, 1997; Wagner, 2010). It has five instar phases, all of which generate vibrations (Bowen et al., 2008; Turchen and Yack, 2025). Recent work has shown that the tiny neonates (∼1–2 mm in length) defend small leaf-tip territories using complex vibratory signals – buzz scrapes and drums – produced during territorial interactions with conspecifics (Matheson et al., 2025). These signals are effective in deterring conspecific intruders and resolving contests without physical aggression. However, these results raise interesting questions about the roles of vibratory detection and signalling within the broader vibroscape. For example, are these complex signals also directed at predators? If so, what is the message being communicated? Also, do caterpillars use vibrations to detect predators to avoid predation, and if so, can they differentiate between vibration sources?

In this study, we investigated the role of vibratory signals in predator–prey interactions in *F. bilineata* caterpillars. We tested three hypotheses: (H1) vibrations produced by territorial neonates function to deter predators; (H2) caterpillars discriminate between predators and conspecific intruders; and (H3) caterpillars use vibrations to discriminate between sources (if H2 is supported). To test these hypotheses, we staged encounters between resident caterpillars and three biologically relevant intruder types: adult ladybird beetles, ladybird beetle larvae and conspecifics.

## MATERIALS AND METHODS

### Insect rearing and collection

Warty birch caterpillars were reared from eggs laid by wild-caught or first-generation females of the two-lined hook tip moth, *Falcaria bilineata* (Packard 1864). Moths were collected at ultraviolet-emitting lights at the Queen's University Biological Station (Chaffey's Lock, ON, Canada; 44.5788°N, 76.3195°W) and various locations near Ottawa, ON, Canada (45.4215°N, 75.6972°W) between May and June 2023, and June and July 2024, or from Howe Bay (Prince Edward Island, Canada; 46.302770°N, 62.408695°W) in August 2009. Gravid females were placed in glass jars, where they laid their eggs on paper birch (*Betula papyrifera*) leaves and twigs, paper or the sides of the glass jar. Upon hatching, neonate larvae were transferred onto paper birch leaves in circular PVC transparent dishes (diameter 5.5 cm, height 1.5 cm) or birch cuttings. Second-generation caterpillars were obtained from couplings of adults that emerged from overwintering pupae. Only first-instar caterpillars, no older than 3 days post-hatching, were used in this study.

Adult convergent ladybird beetles (*Hippodamia convergens*) were purchased from Natural Insect Control (Stevensville, ON, Canada) and used as a predatory species. Adults were maintained in groups of 30 in circular plastic containers (diameter 13.5 cm, height 12.5 cm) lined with absorbent paper and containing birch twigs and leaves. They were fed daily with *ad libitum* access to soaked raisins and drops of honey (Sarwar, 2016), and the containers were misted with water every 2 days. This species is widely distributed in North America (Gordon, 1985) and is one of the most active carnivorous enemies of aphids, including those found on alders (Pergande, 1912). In addition to aphids, *H. convergens* has been shown to feed on lepidopteran eggs (Putman, 1957). Neonate larvae were confirmed to be palatable to these predators, as during our experiments, six individuals were consumed (see Results). Additionally, palatability trials were conducted on *Drepana arcuata*, a species closely related to *F. bilineata* that occurs on the same host plants. *Drepana arcuata* was used for palatability trials because of the limited availability of *F. bilineata* individuals, which were prioritized for experimental trials.

Larvae of the two-spotted ladybird beetle (*Adalia bipunctata*) were obtained as eggs purchased from Natural Insect Control and used as a predatory species. Upon hatching, larvae were reared in isolation in circular PVC transparent dishes (diameter 5.5 cm, height 1.5 cm). Each day, larvae were fed *ad libitum* with soaked raisins, and the dishes were misted with water every 2 days. Only fourth-instar larvae were used for the experiments. This species occurs in North America (Gordon, 1985), and its larvae and adults are active predatory insects on alder and birch (Hajek and Dahlsten, 1987; Skinner and Domaine, 2010). Larvae have been shown to feed on lepidopteran eggs (Putman, 1957). Additionally, palatability trials were conducted on *D. arcuata* caterpillars (see above).

### Behavioural experiments

#### Recording set up

**Fig. 1.**
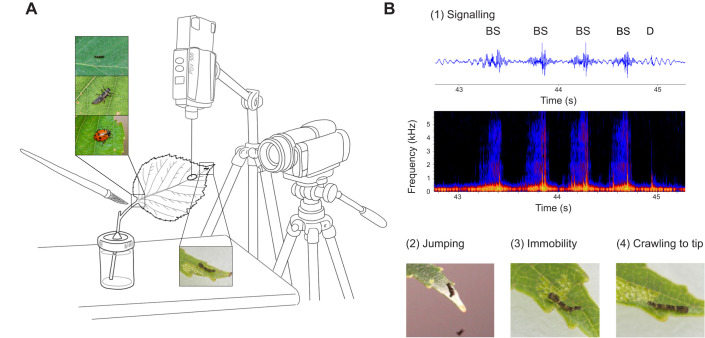
**Overview of experimental setup and caterpillar behavioural responses.** (A) Experimental set up showing the three intruders on the top left and the resident caterpillar at the leaf tip within its territory (i.e. the area marked by feeding scars and a silk mat). The stand holding the vial and the Fostex data recorder are not shown. (B) Main behavioural responses of resident caterpillars following the placement of an intruder on the leaf. (1) Signalling: vibratory signals produced by *Falcaria bilineata* caterpillars. Five signals are represented: four buzz scrapes (BS) and one drum (D) are shown on the waveform (top panel) and spectrogram (bottom panel). (2) Jumping. (3) Remaining motionless. (4) Crawling to the tip of the leaf.

Behavioural interactions between a resident caterpillar and an intruder (predator or conspecific) were conducted using simultaneous vibrational and video recordings (Fig. 1). A water-filled plastic vial containing a birch twig and a single leaf occupied by an established larva (see ‘Predator encounters’, below) was secured to a stable stand. Vibratory data were collected using a laser-Doppler vibrometer (PVD-100; Polytec, Inc., Baden-Württemberg, Germany) (velocity 20 mm s^−1^, HP filter off, LP filter 20 kHz) by focusing the laser beam on a 5 mm diameter 3M Scotchlite^®^ reflective tape attached to the leaf's upper surface, positioned 2–3 cm from the larva. The laser output was connected to a data recorder (Fostex FR-2, Gardena, CA, USA) (sampling rate 48 kHz) and recordings were stored as .wav files. A video camera (Canon XA11 or VIXIA HF G20, Canon Corp., Tokyo, Japan) was focused on the leaf, and vibrations were monitored by connecting the laser output to the microphone port of the camera. A macro lens (Raynox DCR-150, Yoshida Industry Co., Tokyo, Kanto, Japan) was sometimes added for a closer view of interactions. All experiments were performed on an antivibration platform, inside an acoustic chamber (model C-14A MR, Eckel Industries Ltd, Ayer, MA, USA).

#### Predator encounters

Encounters were staged between an established resident larva and an individual predator. In preparation for a trial, a single caterpillar was carefully transferred to the centre of a fresh paper birch leaf (8.4±1 cm in length) using a fine-tipped paintbrush. The leaf's twig was inserted into the lid of a plastic vial (height 6.5 cm) filled with water (Fig. 1A), and a small piece of adhesive putty was applied where the stem entered the hole in the lid to prevent the caterpillar from crawling into the water and to stabilize the twig. The vial was placed inside a mesh enclosure (length 60 cm, width 40 cm, height 40 cm), which was regularly misted. Following a 24 h period, successfully established larvae, evidenced by a visible feeding scar and a silk mat, were selected for trials. Prior to conducting trials, all predators were food deprived for 24 h to standardize hunger levels and weighed.

Each trial consisted of three stages. (1) Undisturbed phase: a vial containing a leaf with a single established caterpillar (the resident) was placed in the recording setup (see above). Following a 1 min acclimation period after placement in the clamp, the resident was recorded undisturbed for 15 min. (2) Predator introduction: a predator was carefully placed onto the leaf using a fine paintbrush. Adult ladybird beetles were placed on the leaf's petiole, while ladybird beetle larvae were mostly placed on the petiole but, because of handling difficulties, occasionally on the centre of the leaf. (3) Interaction phase: the predator was allowed to interact with the resident caterpillar for up to 30 min or until the caterpillar was consumed. Depending on predator behaviour, stages 2 and 3 could occur once or be repeated multiple times within a single trial.

During the interaction phase for each trial, the predator might remain on the leaf for the entire trial, move between the leaf and the vial, or leave the leaf entirely. This sequence of predator presence and absence within the interaction phase is referred to as a cycle. If the predator permanently left the leaf during the 30 min recording period, that individual was removed but the resident caterpillar remained on the leaf. Following a minimum of 5 min or once the resident caterpillar resumed normal activity (e.g. feeding or laying silk), another predator was introduced. The total duration of the interaction phase was fixed at about 30 min per trial, regardless of the number of cycles or number of predators introduced. Therefore, depending on predator behaviour, a resident caterpillar could experience a single continuous predator presence (one cycle) to multiple cycles within a trial. The total time a resident was exposed to predators per trial varied from a few minutes to the full 30 min if the predator never left the leaf, or if the resident was consumed. For adult ladybird beetles, each cycle involved the introduction of a single new individual, and as each trial comprised between one and five cycles, the number of different adult ladybird beetles per trial ranged from one to five. A total of 14 trials were conducted using adult ladybird beetles, involving 39 unique individuals overall. Adults were never reused across trials. For ladybird beetle larvae, the procedure was different because of the limited availability of predators. Within each trial, a single ladybird beetle larva was used for all cycles during the 30 min encounter phase. If the larva left the leaf and had to be re-introduced, the same individual was used. In total, 8 individual ladybird beetle larvae were used across 13 trials, meaning some larvae were reused in multiple trials but they were always paired with a different resident caterpillar.

#### Conspecific encounters

Encounters were staged between a resident larva and a conspecific. The recording set up was the same for the predator trials, described above, except the intruder was not food deprived prior to being placed on the leaf. Because of their small size, neonates were weighed in groups of 10, providing an average mass rather than individual values.

Each trial consisted of three stages. (1) Undisturbed phase: a vial containing a leaf with a single established caterpillar (the resident) was placed in the recording setup (see above) and recorded undisturbed for 15 min. (2) Conspecific introduction: a first-instar conspecific, randomly chosen from the rearing container, was gently transferred onto the centre of the leaf (3– cm from the resident) using a fine-tipped paintbrush. (3) Interaction phase: interactions between the resident and intruder were recorded for up to 30 min. Unlike predator trials, conspecific intruders remained on the leaf for the entire interaction phase, resulting in a single continuous cycle per trial (i.e. no repeated introduction–interaction cycles). Eighteen encounters (trials) were conducted. Note that data collected for this experiment, specifically vibratory signalling rates, were reported in a previous study focusing on territorial encounters between conspecifics (Matheson et al., 2025). Here, we present new analyses on jumping rates and immobility duration from these videos to compare responses of resident larvae to different intruder types.

#### Behavioural analyses

Videos were scored and the data analysed to test the first 2 hypotheses: (H1) vibrations produced by territorial neonates function to deter predators; and (H2) caterpillars discriminate between predators and conspecific intruders.

Video recordings from each trial were scored using BORIS software (v8.27.10; Friard and Gamba, 2016) to quantify the occurrence and timing of both resident and intruder behaviours. For comparative analyses, we focused on specific phases within each trial. These included the 15 min undisturbed phase of the resident caterpillar before the intruder was introduced, and the interaction phase while the intruder was present on the leaf. The interaction phase was subdivided based on the intruder's position relative to the resident's territory: the intruder-on-leaf phase, when the intruder was on the leaf but outside the resident caterpillar's territory, and the intruder-on-territory phase, when the intruder entered the resident's territory. The territory was defined following Matheson et al. (2025) as the area surrounding the leaf tip where the caterpillar had created feeding scars and a silk mat, indicating active occupancy (Fig. 1A). Behaviours of the resident were not scored during the interaction phase when the intruder was absent from the leaf (e.g. when it flew off or moved onto the petiole). Throughout each trial, any caterpillar mortality resulting from interactions was systematically recorded.

To test whether vibratory signalling functions as an antipredator defence (H1), we examined whether resident caterpillars produced vibratory signals in response to intruders, whether signalling rate escalated with intruder proximity and whether intruders responded to these signals by altering their behaviour. Two types of vibratory signal were recorded: drums and buzz scrapes (Fig. 1B; see also Matheson et al., 2025). Drums consisted of rapid, repeated impacts of the anterior body (head, thorax or both) against the leaf surface, producing brief percussive vibrations. Buzz scrapes were more complex signals, generated by a combination of body tremulation and stridulation, often accompanied by percussion from the inclusion of one or several drums at the end of the signal. During each experimental phase (undisturbed, intruder-on-leaf and intruder-on-territory), the number of signalling events produced by the resident caterpillar was recorded, and the signalling rate was calculated as the number of signals divided by the phase duration. Signalling rates were compared across the three experimental phases to assess whether signalling increased as an intruder approached a resident. To determine whether predators responded to resident signals, a key expectation if signalling functions as a predator deterrent, we examined predator behaviour when residents signalled. For trials in which a resident caterpillar produced vibratory signals, we analysed the predator's behaviour in a defined temporal window extending from 2 s before to 2 s after the onset of the resident's signalling bout. This allowed us to detect any immediate behavioural changes in the predator (e.g. stopping, changing direction, retreating) potentially triggered by the caterpillar's signalling.

To test whether caterpillars discriminate between intruder types (H2), we assessed whether residents responded to intruders, how they did so and whether these responses differed among intruder types. If caterpillars discriminate among intruders, we expected them to adjust their defensive strategies according to the perceived level of threat. Beyond vibratory signalling (described above for H1), we identified and scored three additional defensive behaviours exhibited by residents. First, we noted instances of crawling towards the leaf tip, which was only used to classify the type of first defensive response (see below). Second, we measured immobility, the total duration that caterpillars remained completely still, either on the leaf surface or hanging from a silk thread after jumping. Immobility excluded time spent feeding, crawling, laying silk or producing vibratory signals. For each experimental phase, cumulative immobility time was calculated and expressed as a percentage of that phase's duration. Third, we recorded jumping events, defined as sudden detachment from the leaf followed by suspension on a silk lifeline. We calculated jumping rates (jumps per minute) for each experimental phase. Because caterpillars could jump more than once during a trial, for instance when predators moved on and off the leaf across successive cycles, each jump was scored individually. For predator trials, we recorded additional parameters following each jump: total hanging duration (time from jumping off until returning to the leaf), whether the predator was still present when the caterpillar returned to the leaf, the latency (in seconds) before the caterpillar climbed back onto the leaf, and whether the predator was still present upon the caterpillar's return. When caterpillars returned while the predator remained on the leaf, we recorded the predator's location and behaviour to identify the conditions under which residents resumed leaf occupation despite ongoing predator presence. These measurements related to post-jumping behaviours were not taken during conspecific trials because the available video data did not allow reliable tracking of off-leaf periods and returns.

To determine whether residents exhibited defensive responses upon intruder presence, we compared signalling rates, jumping rates and immobility duration between the undisturbed phase and the interaction phases, specifically distinguishing intruder-on-leaf and intruder-on-territory subphases. An increase in any of these behaviours during the interaction phases compared with the undisturbed phase would indicate a defensive response to the intruder. To test whether residents discriminated among intruder types, we compared signalling rates, jumping rates and immobility duration among the three intruder types (conspecifics, ladybird beetle larvae, adult ladybird beetles) within each experimental phase. We also examined whether behavioural responses escalated differently depending on intruder type as intruders moved from outside the territory (intruder-on-leaf phase) to inside the territory (intruder-on-territory phase).

To test whether caterpillars adjust their defensive strategies depending on predator type, we analysed trials in which residents were exposed to predators. If caterpillars perceive different predators as posing different levels of threat, they should respond more quickly to higher-risk predators. To test this, we recorded the latency (in seconds) to the first defensive response and the distance (in cm) between the caterpillar and the predator at that moment. Distances were obtained from video screenshots taken at the first behavioural change, using the 5 mm reflective disc attached to the leaf as a reference to convert pixel measurements into centimetres. Latency was defined as the time elapsed between the predator's placement on the leaf and the first observable change in the resident's behaviour. We assessed behavioural changes by comparing resident behaviour immediately before and after predator introduction. Caterpillars that showed no change in behaviour (e.g. were already immobile prior to the predator's introduction and remained immobile or continued the same activity such as feeding or crawling) were noted but not included in latency analyses. This ensures that the latency and type of first response reflect behavioural change triggered by the intruder. For each caterpillar that responded, we classified the type of first defensive response to assess whether residents employed different defensive strategies depending on predator type. We grouped responses into three functional categories: (1) avoidance, corresponding to immobility; (2) escape, which included crawling toward the leaf tip in preparation for jumping, and/or actual jumping off the leaf and (3) vibratory signalling, previously suggested to act as a deterrent (Matheson et al., 2025). Caterpillars that moved to the leaf tip and then jumped were classified based on whichever behaviour occurred first. These classifications reflect common anti-predator strategies in insects that reduce the risk of predation by avoiding detection, escaping once detected or deterring attacks (Edmunds, 1974).

#### Vibratory detection and discrimination

To determine whether resident larvae use vibratory cues to detect, assess and respond differently to intruder types (H3), we examined vibratory events, and defensive behaviours of resident larvae both before and after jumping.

To assess whether residents might respond to intruder vibrations while residents were on the leaf (i.e. prior to jumping), we first predicted that each intruder type (conspecific, ladybird beetle larva, adult ladybird beetle) generates leaf-borne vibrations while moving on the leaf, and that these vibrations differ in their physical features. For each trial, we selected a 1 s segment of crawling that met the following criteria to allow for standardized comparisons: the segment was uninterrupted (with no pauses), the intruder was crawling across the leaf surface (not on the leaf edge) and the segment was recorded 1–2 cm from the laser. Only trials meeting these criteria were included in the analysis, resulting in sample sizes of *N*=9 for conspecifics, *N*=11 for ladybird beetle larvae and *N*=14 for adult ladybird beetles. Each segment was analysed by measuring several amplitude, spectral and temporal parameters (see Results for visualization of these parameters). The root mean square amplitude (RMS) was calculated from the waveform to capture overall vibration intensity. Spectral parameters were extracted from the power spectrum (8192-point FFT, Hann window, 50% overlap) and included the frequencies of the first and second peaks (Hz) as well as the bandwidth (Hz) at −30 dB from the first peak. Amplitudes of the first and second peaks (dB) were also recorded as measures of relative power at these frequencies. Amplitude and spectral analyses were conducted using Raven Pro (v.1.2; Cornell Laboratory of Ornithology, Ithaca, NY, USA). Temporal characteristics (number of pulses and mean inter-pulse interval), were analysed using Avisoft-SASLab Pro (v.1.5.22; Avisoft Bioacoustics, Berlin, Germany). These acoustic parameters were then compared among the three intruder types (conspecifics, ladybird beetle larvae, adult ladybird beetles) to determine whether each group produced a distinct vibrational profile (see below). Second, we predicted that if resident caterpillars use leaf-borne vibrations to detect and assess intruders, they may sometimes initiate a defensive response prior to being physically contacted by the intruder. To test this, video recordings were reviewed to identify instances in which residents initiated defensive responses (immobility, jumping, crawling to the leaf tip or vibratory signalling) while the intruder was still at a measurable distance. The distance at the moment of first defensive response (recorded for predator encounters, see H2 above) provided evidence for detection before contact. Third, we tested whether resident larvae might also use vibrations to assess the risk of returning to the leaf following a jump. In trials where caterpillars jumped, we examined whether they returned while the predator was still present and, if so, under what circumstances (e.g. predator motionless or having moved away). If caterpillars predominantly returned only after vibrations ceased, this would support the hypothesis that they monitor predator presence via substrate-borne vibrations transmitted through the silk lifeline.

### Statistical analyses

Because the signalling rate of resident caterpillars did not meet model assumptions, a robust non-parametric approach, the aligned rank transform (ART) ANOVA (function *art* from the package *ARTool*; Wobbrock et al., 2011), was used to analyse the effects of intruder type and the experimental phase (defined here as the behavioural context experienced by the resident: resident undisturbed, intruder on the leaf but outside the territory, and intruder on the territory) on the signalling rate of resident caterpillars. When significant effects of factors or interactions were detected, pairwise comparisons were conducted using contrasts on ART-transformed data (function *art.con* from *ARTool*), with *P*-values adjusted for multiple comparisons using Tukey's method. The same approach was also used to assess the effects of intruder type and experimental phase on the jumping rate of resident caterpillars, and the percentage of time spent immobile. We compared the number of resident caterpillars that were killed by a predator during trials between the two predator types (adult ladybird beetle versus ladybird beetle larvae) using log-rank tests (function *survdiff* from the *survival* package: https://CRAN.R-project.org/package=survival). We used non-parametric Wilcoxon rank-sum tests both to compare the latency of the resident caterpillar's first behavioural response following predator introduction and to compare the predator–resident distance at the moment behavioural changes occurred. Finally, to assess whether different intruder types produced distinct vibrational signatures during crawling, we conducted a principal component analysis (PCA) on eight vibrational parameters: RMS, first and second peak frequencies and their relative amplitudes, bandwidth of first peak, number of pulses and mean inter-pulse interval. The PCA was performed using the *FactoMineR* package (Lê et al., 2008), and individual observations were plotted in the space of the first two principal components (PC1 and PC2), which together explained the majority of variance. To compare the multivariate outputs of the PCA, a multivariate analysis of variance (MANOVA) was conducted using the individual scores of the first two principal components (PC1 and PC2) as dependent variables and the ‘intruder type’ as the explanatory factor. Following a significant MANOVA, *post hoc* differences between groups were assessed using Tukey's method on each principal axis separately. All statistical analyses were performed in R v.4.4.2 (http://www.r-project.org/).

## RESULTS

This study tested three hypotheses: (H1) vibrations produced by territorial neonates function to deter predators; (H2) caterpillars discriminate between predators and conspecific intruders; and (H3) caterpillars use vibrations to discriminate between intruders. We staged encounters between resident caterpillars and three intruder types: conspecifics, adult ladybird beetles and ladybird beetle larvae. If H1 is supported, residents should signal to predators and predators should respond to these signals. If H2 is supported, defensive responses should vary by intruder type. If H3 is supported, each intruder type should produce distinct vibrational signatures.

### Vibratory signalling in response to intruders

**Fig. 2.**
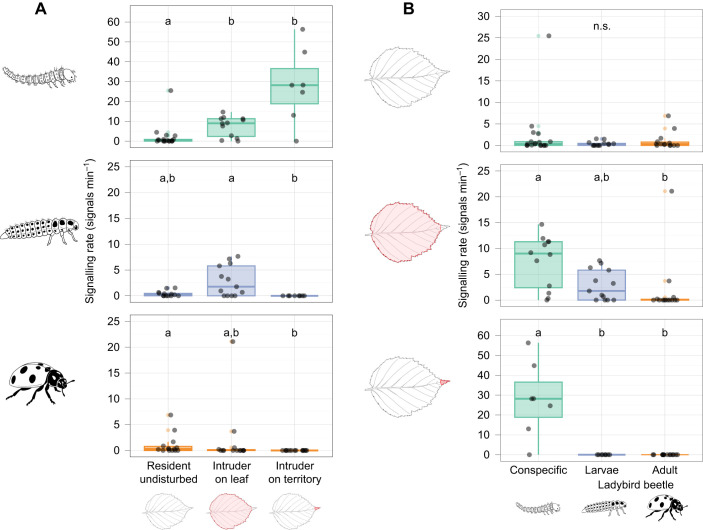
**Boxplots showing the resident caterpillar signalling rate according to intruder type and experimental phase.** (A) Type of intruder: a conspecific, a ladybird beetle larva and an adult ladybird beetle. (B) Experimental phase (depicted by leaf shading): when the resident is undisturbed, when an intruder is on the leaf and when the intruder is on the territory. Each grey dot represents one observed value. Data include 18 conspecific trials (though only 13 intruders were on the leaf and 7 entered the territory), 13 ladybird beetle larva trials and 14 adult ladybird beetle trials. The horizontal line in each box represents the median and the lower and upper hinges indicate the first and third quartiles. Lower and higher whiskers extend to the most extreme values within 1.5 interquartile ranges from the first and third quartiles, respectively. Coloured dots indicate outliers.

The first hypothesis tested whether vibratory signalling in territorial warty birch caterpillars functioned to deter predators. Resident larvae responded to all three intruder types by signalling but modulated their signalling rates depending on intruder type and stage of encounter (Fig. 2).

Signalling rate of residents varied according to both the type of intruder (ART: *F*_2,104_=44.54, *P<*0.001) and the stage of the encounter as it approached the resident (ART: *F*_2,104_=28.51, *P*<0.001), the interaction between the two being significant (ART: *F*_4,104_=25.84, *P*<0.001; Fig. 2).

Resident caterpillars exhibited distinct signalling patterns depending on intruder type. In response to conspecific intruders, signal rates escalated dramatically as the intruder approached, increasing from a baseline of 2.18±5.95 signals min^−1^ during the undisturbed phase to 7.49±5.07 signals min^−1^ when the intruder was on the leaf (Tukey's test: *P*=0.024), and peaking at 27.9±18.74 signals min^−1^ once the intruder entered the resident's territory (Tukey's test: *P*<0.01; Fig. 2A; Movie 1). In response to ladybird beetle larvae, signalling increased slightly when the intruder was introduced, but then dropped completely to zero as the intruder entered the territory. There was a non-significant increase of the signalling rate during the on-leaf phase (2.86±2.95 signals min^−1^) compared with baseline (0.39±0.54 signals min^−1^), followed by a significant drop to zero when the larva entered the territory (Tukey's test: *P*<0.01; Fig. 2A; Movie 2). In response to adult ladybird beetles, resident caterpillars were largely silent throughout the on-leaf and on-territory phases. Signalling rates started at 1.07±1.97 signals min^−1^ during the undisturbed phase, increased slightly to 1.82±5.63 signals min^−1^ while the predator was on the leaf, and then significantly decreased to 0 signals min^−1^ when the predator entered the territory (Tukey's test: *P*=0.038; Fig. 2A).

We also assessed whether resident larvae signalling rates differed within a particular phase depending on which intruder was presented. As expected, there were no differences in resident signalling prior to introducing an intruder (i.e. during the undisturbed phase) (Fig. 2B). However, during the intruder-on-leaf phase, signalling rate was significantly higher in response to conspecifics (7.49±5.07 signals min^−1^) compared with adult ladybird beetles (1.82±5.63 signals min^−1^; Tukey's test: *P*<0.001), with ladybird beetle larvae eliciting intermediate rates (2.86±2.95 signals min^−1^; Fig. 2B; Movies 1–3). In the on-territory phase, signalling rate was significantly higher in response to conspecifics than for both adult ladybird beetles (Tukey's test: *P*<0.001) and ladybird beetle larvae (Tukey's test: *P*<0.001; Fig. 2B; Movies 1–3), both of which elicited no signalling.

Although signalling in response to predators was minimal, in cases where resident larvae did signal, we examined predator responses. We found that predator responses to resident signals were minimal. During the on-leaf phase (the only phase where residents signalled to predators), a total of eight signalling bouts were recorded in the presence of adult ladybird beetles, and 131 bouts in the presence of ladybird beetle larvae (excluding 24 bouts where the predator was out of view). Adult ladybird beetles continued walking in most cases (6/8 bouts), with rare instances of stopping, local searching or predation. Ladybird beetle larvae mostly remained motionless (*n*=39) or continued walking (*n*=55), with occasional changes in direction (*n*=9) or brief starts/stops of movement (*n*=3).

In summary, resident caterpillars produced strong vibratory responses to conspecific intruders, particularly when intruders entered their territory, while signalling in response to predators was infrequent. Predators showed little or no reaction to these signals, indicating that vibratory signalling functions primarily as a territorial behaviour rather than an antipredator strategy.

### Jumping behaviour in response to intruders

**Fig. 3.**
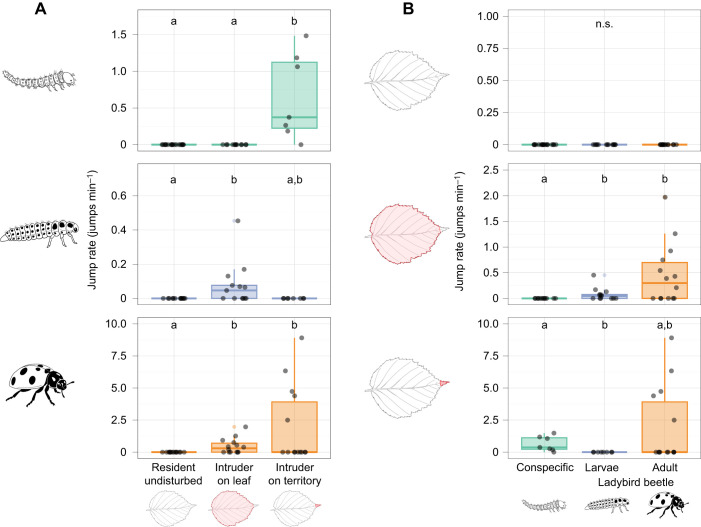
**Boxplots showing the resident jumping rate according to intruder type and experimental phase.** (A) Type of intruder: a conspecific, a ladybird beetle larva and an adult ladybird beetle. (B) Experimental phase (depicted by leaf shading): when the resident is undisturbed, when an intruder is on the leaf and when the intruder is on the territory. Each grey dot represents one observed value. Data include 18 conspecific trials (though only 13 intruders were on the leaf and 7 entered the territory), 13 ladybird beetle larva trials and 14 adult ladybird beetle trials. The horizontal line in each box represents the median and the lower and upper hinges indicate the first and third quartiles. Lower and higher whiskers extend to the most extreme values within 1.5 interquartile ranges from the first and third quartiles, respectively. Coloured dots indicate outliers. Note that *y*-axes differ between panels to better visualize the data distribution for each species.

Resident larvae jumped in the presence of all intruder types, but mostly in response to adult ladybird beetles (Fig. 3A). Jumping involved a sudden detachment from the leaf tip, followed by suspension from a silken lifeline attached to the leaf. Jumping rates were significantly affected by both experimental phase (ART: *F*_2,104_=29.31, *P*<0.001) and intruder type (ART: *F*_2,104_=34.85, *P*<0.001), with a significant interaction between the two terms (ART: *F*_4,104_=11.26, *P*<0.001; Fig. 3).

Jumping patterns varied markedly depending on intruder type and timing of physical contact. Resident larvae responded to conspecifics by jumping only when the intruding larva entered the territory (0.65±0.55 jumps min^−1^; Tukey's test: *P*<0.001; Fig. 3A; Movie 1), and always upon physical contact with the resident. In contrast, jumps in response to predators occurred without physical contact. Ladybird beetle larvae elicited a low jumping rate during the on-leaf phase (0.08±0.13 jumps min^−1^), which represented a significant increase compared with the undisturbed phase (Tukey's test: *P*=0.018; Fig. 3A; Movie 2), but no jumps were recorded during the territory phase. Adult ladybird beetles triggered 0.46±0.59 jumps min^−1^ in the on-leaf phase, significantly higher than in the undisturbed phase (Tukey's test: *P*<0.001), followed by a further increase to 1.92±2.98 jumps min^−1^ in the on-territory phase (Tukey's test: *P*=0.026; Fig. 3A; Movie 3). No jumping was observed during the undisturbed phase in any trial.

Within a particular phase, jumping differed depending on the intruder type. During the on-leaf phase, jumping rate was significantly higher in the presence of adult ladybird beetles (Tukey's test: *P*<0.001) and ladybird beetle larvae (Tukey's test: *P*=0.022; Fig. 3B) than with conspecifics. In the territory phase, jumping rate was highest in response to adult ladybird beetles, followed by conspecifics, which elicited a significantly higher jumping rate than ladybird beetle larvae (Tukey's test: *P*<0.001), but did not differ significantly from adult ladybird beetles (Fig. 3B).

In summary, jumping behaviour differed sharply depending on intruder type: with predators, jumps occurred early, when the intruder was on the leaf but not in the larval territory, whereas with conspecifics, jumps occurred only upon physical contact in the territory.

#### Return to the leaf following jumps

Following a jump, a resident caterpillar may return to the leaf by crawling back onto the leaf by its silk lifeline during the course of the trial. In most cases, caterpillars only returned to the leaf if the predator had departed or had become motionless, and this return behaviour differed depending on the type of predator. After interactions with adult ladybird beetles, most caterpillars (95.45%, *N*=21/22) climbed back onto the leaf after the predator had departed, with a mean latency of 47.22±40.53 s following departure (Movie 3). In contrast, only 60% (*N*=9/15) returned after the departure of a ladybird beetle larva, with a longer mean latency of 65.54±62.28 s following departure (Movie 2). Returning to the leaf while the predator was still present occurred only when the predator became completely motionless. Trials with conspecific intruders were not monitored beyond the initial interaction phase, so comparable return data were not collected for this treatment.

These results show that resident caterpillars only return when predators have left the leaf or have become immobile, and the decision to return is dependent upon which predator they were exposed to. This suggests that suspended larvae use vibratory cues to assess when it is safe to return to the leaf (see further discussion below under ‘Vibration-mediated predator avoidance’).

### Immobility

**Fig. 4.**
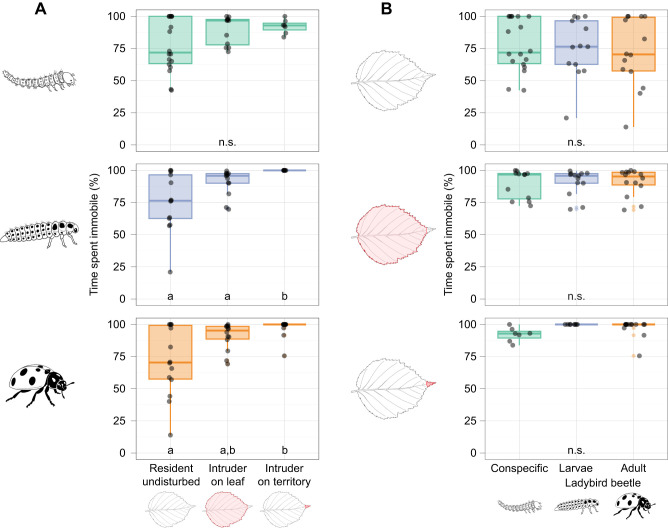
**Boxplots showing the percentage of time spent motionless by a resident according to intruder type and experimental phase.** (A) Type of intruder: a conspecific, a ladybird beetle larva and an adult ladybird beetle. (B) Experimental phase (depicted by leaf shading): when the resident is undisturbed, when an intruder is on the leaf and when the intruder is on the territory. Each grey dot represents one observed value. Data include 18 conspecific trials (though only 13 intruders were on the leaf and 7 entered the territory), 13 ladybird beetle larva trials and 14 adult ladybird beetle trials. The horizontal line in each box represents the median and the lower and upper hinges indicate the first and third quartiles. Lower and higher whiskers extend to the most extreme values within 1.5 interquartile ranges from the first and third quartiles, respectively. Coloured dots indicate outliers.

Immobility of the resident caterpillar increased from baseline levels in response to all intruder types, and continued to increase as the intruder approached the territory (Fig. 4). Immobility differed significantly across phases (ART: *F*_2,104_=10.01, *P*<0.001), but not according to intruder type (ART: *F*_2,104_=0.55, *P*=0.57), nor was there a significant interaction between the two terms (ART: *F*_4,104_=0.46, *P*=0.76; Fig. 4).

Resident caterpillars increased their immobility as a conspecific moved from the leaf to the territory although this trend was not significant (Fig. 4A). When exposed to ladybird beetle larvae, immobility increased gradually, reaching complete immobility during the on-territory phase (undisturbed versus on-territory: 75.15±23.05% versus 100%, Tukey's test: *P*=0.023; on-leaf versus on-territory: 90.62±10.11% versus 100%, *P*<0.001; Fig. 4A). In the presence of adult ladybird beetles, immobility was also higher during the territory phase (97.46±6.70%) compared with the undisturbed phase (71.45±27%; *P*=0.002; Fig. 4A).

Within a given phase there were no significant differences in resident immobility between intruder types (Fig. 4B). However, when the intruder was on the territory, immobility tended to be slightly lower in the presence of conspecifics than predators. This pattern likely reflects the high rate of vibratory signalling directed at conspecifics during this phase (Fig. 2), whereas residents remained silent and immobile in response to predators.

Overall, immobility of residents increased from baseline activity levels across all phases, particularly in response to predators. Increased immobility as a predator approaches could indicate that residents are exhibiting acoustic crypsis and/or are ‘listening’ to assess risk.

### Antipredator strategies and survival of resident larvae

To better understand the antipredator strategies of resident larvae, we also assessed how a resident first responded in the presence of a predator, whether the larva survived the encounter and, if so, what it did after the predator left.

#### First defensive response to predators

**Fig. 5.**
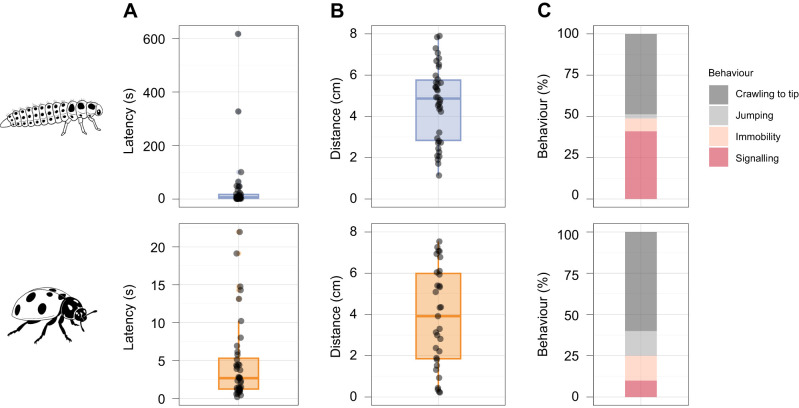
**First behavioural responses of a resident larva in the presence of a predator.** (A) Latency before the first behavioural response of resident caterpillars following the placement of a predator on the leaf. Note that the *y*-axis scales differ between predator types to best represent the observed range of latencies. (B) Distance between the predator and the resident caterpillar at the moment the caterpillar changed its behaviour. (C) Proportion of first behavioural responses of resident caterpillars depending on predator type. Data shown are from 13 trials with ladybird beetle larvae and 14 trials with adult ladybird beetles, yielding 40 predator–caterpillar interactions per predator type. Each grey dot represents one observed value, and coloured dots indicate outliers.

Resident caterpillars adjust their first defensive strategy depending on the predator encountered. To quantify these responses, we recorded the latency to the first behavioural change and the distance to the predator at which it occurred (Fig. 5), considering only instances where the resident caterpillar changed its behaviour after introduction of the predator.

The latency to the first behavioural response of the resident caterpillar following the introduction of a predator on the leaf differed significantly between the two predator types, with faster (lower latency) responses to adult ladybird beetles (4.59±5.26 s; *N*=40; Movie 3) than to ladybird beetle larvae (37.6±108 s; *N*=40; Wilcoxon test: *W*=1179.5, *P<*0.001; Fig. 5A). This highlights that caterpillars detect and/or respond to adult ladybird beetles more readily than to ladybird beetle larvae.

The distance between the resident caterpillar and the predator at the time of the first behavioural response did not differ significantly between predator types. Caterpillars were at similar distances from ladybird beetle larvae (4.81±1.95 cm; *N*=40) and adult ladybird beetles (5.02±3.02 cm; *N*=40; Wilcoxon test: *W*=755, *P*=0.67; Fig. 5B; Movie 3), indicating that the spatial proximity to the predator at the onset of response was comparable regardless of predator type.

The type of initial behavioural response upon intruder presence varied with predator type (Fig. 5C). Crawling to the tip and jumping were the most frequent initial responses overall and were proportionally more common in response to adult ladybird beetles (75%; Movie 3) than to ladybird beetle larvae (51.3%; Fig. 5C). In contrast, vibratory signalling occurred more frequently in response to ladybird beetle larvae (41%) than to adults (10%; Fig. 5C), while immobility was the least frequent first response overall (larvae: 7.7%; adults: 15%; Fig. 5C). These results indicate that caterpillars shift their immediate defensive strategy depending on the predator they encounter.

Together, these results suggest that caterpillars flexibly tailor their immediate defensive behaviours according to predator type as it approaches. We propose that these decisions are mediated by substrate-borne vibrations (see ‘Vibration-mediated predator avoidance’, below).

#### Caterpillar survival

Caterpillars faced a higher risk of mortality when exposed to adult ladybird beetles than to ladybird beetle larvae, indicating that adult ladybird beetles represent a greater threat. Survival analyses revealed higher mortality risk in the presence of adult ladybird beetles. None of the caterpillars exposed to ladybird beetle larvae died (*N*=13), whereas 43% of individuals died during trials involving adult ladybird beetles (6 deaths over 14 trials, log-rank test: χ^2^_1_=7.2, *P*=0.007; Movie 3). In all cases, adult ladybird beetles approached and attacked caterpillars by foot.

Such variation in risk may underlie the differences observed in behavioural responses of the resident. Resident caterpillars responded more rapidly and with more escape behaviours to adult ladybird beetles, whereas responses to ladybird beetle larvae were slower and included relatively more vibratory signalling. The survival data thus provide an ecological context for interpreting the adaptive value of these immediate defensive strategies.

#### Post-encounter territory abandonment

Of resident caterpillars that survived the encounter, some abandoned their territory following the departure of the predator. Following encounters with adult ladybird beetles, 5 of 8 surviving individuals (62.5%) abandoned their territory and began wandering across the leaf surface, a behaviour never observed after the final departure of ladybird beetle larvae or during the 15 min undisturbed phase at the start of the trials. This result suggests that exposure to higher-risk predators may influence subsequent spatial behaviour, potentially reflecting a strategy to avoid future encounters.

### Vibration-mediated predator avoidance

Our results provide support for three ways that resident larvae use vibrations in predator avoidance: discrimination, vigilance and acoustic crypsis.

#### Do resident larvae discriminate between intruders based on locomotory vibrations?

Our third hypothesis stated that resident caterpillars use vibrations to discriminate between intruders. We predicted that (1) intruders produce vibrations when approaching the resident on the leaf; (2) that these vibrations are distinct; and (3) that sometimes first responses of residents occur before contact and when predator vibrations are accessible to the resident.

**Fig. 6.**
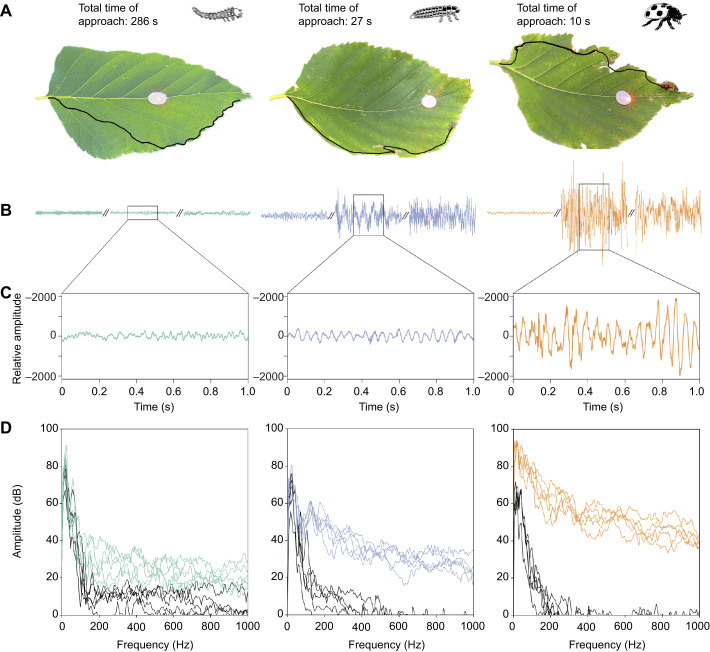
**Crawling vibrations for each intruder type on birch leaves.** (A) Representative pathways of different intruders (conspecific, ladybird beetle larva and ladybird beetle adult) as they approach the resident larva on its leaf-tip territory. (B) Concatenated waveforms showing three consecutive 2 s vibration sequences, one from each experimental phase: resident undisturbed (prior to introducing the intruder), intruder on the leaf and intruder on the territory; amplitude is shown on a relative scale. Double slashes (//) indicate breaks between phases. (C) Expanded waveforms from B, showing 1 s segments of crawling vibrations selected from the intruder-on-territory phase (indicated by boxes in B), recorded at a distance of 1–2 cm from the laser; amplitude is shown on a relative scale. (D) Power spectra for vibrations produced by a crawling intruder. Coloured lines represent five spectra recorded from separate trials using different individuals and leaves. Black lines show five representative spectra of background noise, sampled from the same individuals as the crawling vibrations.

**Fig. 7.**
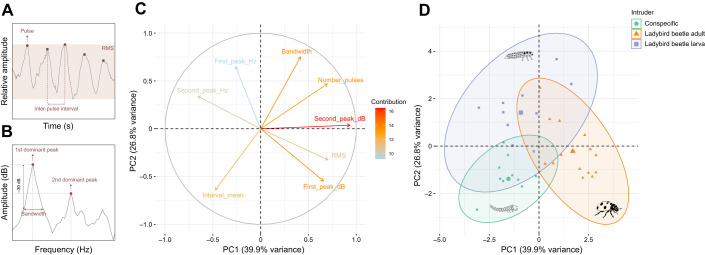
**Principal components analysis (PCA) related to crawling vibrations of three intruder types.** (A) Schematic example of a waveform of a crawling vibration, illustrating the temporal and amplitude parameters measured: number of pulses (red dots), mean inter-pulse interval and root mean square amplitude (RMS) (shaded area). (B) Schematic example of a power spectrum of a crawling vibration, illustrating the spectral and amplitude parameters measured: the peak frequency and relative amplitude of the first and second peaks, as well as the bandwidth (Hz) at −30 dB from the first peak. Axes in A and B are not labelled numerically, as the schemes are provided for illustrative purposes only. (C) Relationships between spectral (First_peak_Hz, Second_peak_Hz, Bandwidth), amplitude (First_peak_dB, Second_peak_dB, RMS) and temporal (Number_pulses, Interval_mean) variables from on-leaf crawling vibrations in the space of PC1 (39.9% variance) and PC2 (28.8% variance). Contributions of each variable are indicated by colour intensity. (D) PCA plots showing clustering of intruder types. Symbols distinguish groups: conspecifics, green circles, *N*=9; ladybird beetle larvae, purple squares, *N*=11; and adult ladybird beetles, orange triangles, *N*=14. The 95% confidence ellipses delineate the groups. The larger dots in the plot indicate the centroid of each group.

Vibrations are produced by all intruder types as a byproduct of crawling (Fig. 6). Analysis of the crawling vibrational sequences revealed distinct vibratory ‘signatures’ for each intruder type based on their spectral, amplitude and temporal characteristics. A PCA was conducted on the eight measured vibrational variables to visualize and quantify differences in crawling sequences across the three intruder types (Fig. 7). Vibrational signatures differed significantly among intruder types (MANOVA: Pillai=1.20, *F*_4,62_=23.33, *P*<0.001). The primary axis (PC1, 39.9% of the variance) is driven by a combination of variables measuring energy (RMS, and relative amplitude of first and second peaks) and temporal events (number of pulses), collectively reflecting overall signal energy and complexity (Fig. 7C). This axis significantly separates adult ladybird beetles from both ladybird beetle larvae and conspecifics (Tukey's test: *P*<0.001 for both), with adult ladybird beetles producing high-energy vibrations with a higher number of pulses, while ladybird beetle larvae and conspecifics produce lower-energy vibrations with fewer pulses (Fig. 7D). This pattern may be partly influenced by the body mass of the intruders: adult ladybird beetles are heavier (19.73±4.46 mg) than ladybird beetle larvae (3±0.84 mg) and conspecifics (0.81±0.2 mg).

The second axis (PC2, 28.8% of the variance) captures variation in the spectral and temporal structure of the vibrations, with strong contributions from bandwidth and the mean inter-pulse interval (Fig. 7C). While conspecifics and ladybird beetle larvae do not differ significantly on the first axis (Tukey's test: *P*=0.42), they are clearly discriminated along this second dimension (Tukey's test: *P*<0.001), differentiating conspecifics from predators. Conspecifics (positioned at the bottom) produce narrow-bandwidth vibrations with longer inter-pulse intervals, indicating that most of the vibrational energy is concentrated within a limited frequency range, suggesting more regular and structured vibrations (Fig. 7D). In contrast, both predator types generate broader-bandwidth vibrations with shorter inter-pulse intervals, reflecting a wider spread of frequencies, pointing to more complex or noisy spectral profiles (Fig. 7D). In summary, the combination of variables measuring energy (which separates adult ladybird beetles from other intruders along PC1), spectral bandwidth (which separates conspecifics from predators along PC2) and temporal characteristics (which contribute to separation along both axes) creates a unique vibrational signature for the crawling of each group.

Caterpillars initiated defensive responses before physical contact with predators, providing evidence for vibratory detection. When residents first changed their behaviour in response to predators, always before any physical contact, they were at a mean distance of 4.81±1.95 cm from ladybird beetle larvae (*N*=40) and 5.02±3.02 cm from adult ladybird beetles (*N*=40; see ‘First defensive response to predators’, above; Fig. 5B). These distances demonstrate that residents detected predators before contact, consistent with detection via substrate-borne vibrations. Given the limited visual capacity of early instars and their position on the leaf, substrate-borne vibrations represent the most parsimonious sensory modality for remote predator detection, although other cues cannot be excluded (see Discussion).

#### Vigilance and vibratory crypsis

Our results suggest that vibrations play additional roles in predator avoidance. First, immobility was the most common response to predators, particularly once predators entered the territory (Fig. 4). During the undisturbed phase, recordings from the leaf showed vibrations associated with feeding, crawling and signalling, but these are absent during immobility. This absence of vibrations when predators are present is consistent with both vibratory vigilance (reducing one's own noise to better detect predator movements) and vibratory crypsis (reducing detectability to predators). Second, as reported above (see ‘Return to the leaf following jumps’), caterpillars suspended on silk threads only returned to the leaf when predators had departed or had become completely motionless. In all cases where caterpillars initiated their return (*N*=37), no vibrations were detected on the leaf at that moment (Movies 2 and 3). This suggests that residents monitor vibrations transmitted through the silk thread to assess risk and predator presence before returning.

## DISCUSSION

Our results reveal a sophisticated system of vibration-mediated intruder detection and context-dependent defensive responses in tiny neonate warty birch caterpillars. Vibrations may play crucial roles in predator defence through early detection, discrimination and crypsis. In contrast, vibratory signalling by resident neonates occurred predominantly in the presence of conspecifics, confirming its role as a territorial display.

### Vibratory signals as territorial rather than antipredator displays

Our first hypothesis, that vibratory signals produced by territorial neonates function to deter predators, was not supported. Resident caterpillars produced strong vibratory responses to conspecific intruders, with signalling rates escalating dramatically as intruders entered their territories. In contrast, signalling ceased when predators approached and entered the territory, with few or no signals produced in response to either adult ladybird beetles or ladybird beetle larvae. These results strongly suggest that vibratory signals of *F. bilineata* neonates evolved primarily to defend leaf territories against conspecifics, as previously shown in this species (Matheson et al., 2025) and other Lepidoptera larvae (Fletcher et al., 2006; Scott et al., 2010; Scott and Yack, 2012; Yack et al., 2001, 2014). The territorial function of these signals is reinforced by the lack of predator responses. Neither adult ladybird beetles nor larvae showed consistent deterrence responses to vibratory signals, with most continuing their approach or remaining motionless. This contrasts with systems where vibratory signals produced by caterpillars play a role in antipredator defence, such as *Antispila nysaefoliella* producing signals that may interfere with parasitoid foraging (Low, 2008), *D. arcuata* generating vibrations that deter stink bug attacks (Guedes et al., 2012), and lycaenid larvae emitting signals that attract protective ants (Travassos and Pierce, 2000). This could suggest that vibratory antipredator signals may be more effective against certain predator types (parasitoids, vertebrates, ants) than against hunting coccinellids, which may lack the sensory mechanisms to detect these signals, do not perceive them as threatening or have evolved to ignore them if they provide no reliable information about prey palatability or defensive capability. However, just because these signals do not deter the predators tested here does not preclude their effectiveness against other natural enemies in *F. bilineata's* predator community, including pentatomid bugs, wasps, mantids or spiders. Additionally, because larvae signal during undisturbed phases, these territorial signals could inadvertently make them detectable to eavesdropping predators or parasitoids. Thus, while vibratory signalling plays a critical role in the ecology of *F. bilineata*, our results indicate that its primary function is mediating territoriality rather than deterring predators such as coccinellids, with other defensive strategies deployed when such predation threats arise.

### Caterpillars discriminate between intruder types

Our second hypothesis, that resident caterpillars discriminate between predators and conspecific intruders, was strongly supported. Residents adjusted their behaviours depending on who was approaching, deploying responses that matched the specific threat level posed by each intruder type. This discrimination was not simply binary (predator versus conspecific), but rather reflected a graded assessment system where defensive responses were finely calibrated to threat level. This sophisticated threat assessment was evident across multiple independent behavioural metrics. When facing conspecifics, residents relied heavily on signalling that escalated as the intruder approached their territories, jumping only when physical contact occurred. Predators, however, triggered a very different defensive repertoire. Residents suppressed all signalling when predators entered their territories, instead prioritizing escape through jumping, and doing so much earlier in the encounter, often before the predator reached the territory. This jumping behaviour represented a key escape mechanism against predators, involving rapid detachment from the leaf followed by suspension from a silk lifeline that effectively removed the caterpillar from immediate danger. Dropping from vegetation is common among caterpillars facing predators or parasitoids (Castellanos et al., 2011; Fitzpatrick et al., 1994; Gentry and Dyer, 2002). However, dropping carries costs, including exposure to ground-dwelling predators, desiccation risk and the energetic expense of relocating to suitable host plants. The use of silk lifelines during dropping, as documented in *F. bilineata* (Matheson et al., 2025) and other species facing predators (Castellanos and Barbosa, 2006), partially mitigates these costs by maintaining a connection to the original leaf and facilitating return once danger passes (Sugiura and Yamazaki, 2006). Discrimination between intruder types was also evident in immobility patterns. Immobility increased as predators approached, reaching 97–100% of time when they were in the territory versus lower values with conspecifics. This behaviour, typically described as a freezing response (Sakai, 2021), likely serves dual functions in predator avoidance. First, remaining motionless may constitute a form of vigilance, allowing caterpillars to focus on detecting and processing vibratory cues from approaching threats without generating masking noise from their own movements. Second, immobility represents a form of acoustic crypsis. Active caterpillars produce vibrations through feeding, crawling and signalling. By ceasing these activities, immobile caterpillars become vibrationally silent, potentially reducing their detectability to vibration-sensitive predators. While we did not test whether ladybird beetles detect prey through vibrations, many predatory insects exploit incidental prey vibrations for localization, including pentatomid stink bugs, antlion larvae and web-building spiders (Pfannenstiel et al., 1995; Devetak, 2014; Barth, 1998). Because predators often use substrate-borne vibratory cues to locate prey, immobility may function as a form of acoustic crypsis. Similar defensive strategies have been observed in other insects: for example, vibratory cues trigger reduced locomotor activity in the weevil *Aegorhinus nodipennis* (Stritih-Peljhan et al., 2025) and freezing behaviour in the leaf-dwelling beetle *Paraglenea fortunei* (Tsubaki et al., 2014).

Beyond distinguishing predators from conspecifics, residents also differentiated between the two predator types themselves, calibrating their responses to match the threat each posed. This finer-scale discrimination aligned with observed mortality rates: adult ladybird beetles killed 43% of caterpillars encountered, whereas ladybird beetle larvae killed none. Residents reacted to adult ladybird beetles nearly eight times faster than to ladybird beetle larvae. When adult ladybird beetles approached, caterpillars typically escaped immediately. In contrast, they more often signalled first when encountering ladybird beetle larvae, as though testing whether territorial displays might work before resorting to escape. Jumping rates followed a similar pattern: adults triggered much higher jumping rates throughout encounters, while larvae elicited relatively few jumps. The starkest difference appeared after encounters ended. Over half the caterpillars that survived adult ladybird beetle attacks abandoned their leaf-tip territories and wandered across the leaf, a behaviour we never observed after encounters with ladybird beetle larvae or conspecifics, where all residents survived and stayed put, or during undisturbed phases prior to intruder introduction. This is particularly striking given the value of leaf-tip territories. Competition for these positions is intense among *F. bilineata* neonates, likely because leaf tips offer multiple advantages: they may provide superior nutritional quality, optimal microclimatic conditions and potentially reduced exposure to predators or parasitoids approaching from the leaf interior (Matheson et al., 2025). Additionally, residents have established silk mats that provide secure footing. Abandoning these established territories means forfeiting these investments and exposing the caterpillar to multiple risks during dispersal, including increased exposure to predators, desiccation and the possibility of failing to locate suitable new feeding sites. Resident caterpillars only abandoned territories after adult ladybird beetle encounters, which were associated with the highest mortality and the highest jumping rates, suggesting they weigh the costs of relocation against the perceived ongoing risk, abandoning only when the threat level justifies the cost.

Our results suggest that resident caterpillars are capable of recognizing the level of threat and responding in accordance with the threat-sensitivity hypothesis (Helfman, 1989). Across all behavioural metrics (signalling rates, response latencies, jumping rates, immobility levels and post-encounter territory decisions), resident caterpillars exhibited a graded response system where defensive intensity scaled with perceived threat of predation: conspecifics (no predation threat)<ladybird beetle larvae (0% mortality)<adult ladybird beetles (43% mortality). These results demonstrate sophisticated risk assessment in resident larvae. What makes these results particularly striking is the fineness of discrimination achieved by animals barely 2 mm long, with limited sensory systems, facing a complex predator community that includes not only coccinellids but also potentially pentatomid bugs, wasps, mantids, ants and spiders (Greeney et al., 2012; Heinrich, 1993). How do these tiny larvae accomplish such precise threat discrimination? Our evidence suggests that this recognition and decision making begin with substrate-borne vibratory cues.

### Evidence for vibration-mediated discrimination

Having established that caterpillars discriminate between intruder types, our third hypothesis, that this discrimination is mediated by substrate-borne vibrations, was supported by three lines of evidence. First, each intruder type produced leaf-borne vibrations with distinct vibratory signatures as they approached the resident caterpillar. Body mass differences likely contributed to both amplitude and spectral variation. Adult ladybird beetles are substantially heavier than caterpillars, and vibrational energy at the source can vary widely depending largely on the size and available muscular power of the emitter (Markl, 1983). In our study, variables related to amplitude and energy appeared to provide reliable cues of threat level, with the highest-energy crawling vibrations produced by the most dangerous predators, adult ladybird beetles. In contrast, overlap in these amplitude-related measures between conspecifics and ladybird beetle larvae may explain why residents sometimes initially responded to ladybird beetle larvae with signalling before switching to escape behaviours. Spectral and temporal features likely provide complementary information. The narrow-band vibrations of conspecifics contrast with the broader-bandwidth signals of predators, potentially reflecting differences in locomotor biomechanics, as shown for caterpillars versus stink bugs (Guedes et al., 2012), and enabling discrimination between territorial and predatory contexts. Temporal characteristics, including pulse number and inter-pulse interval, likely reflect the stepping patterns (‘footsteps’) of the intruders as they move across the leaf. Each pulse can be interpreted as a discrete contact event between the body or appendages and the substrate, meaning that pulse rate and spacing may encode information about locomotor dynamics such as speed, gait and coordination. These temporal features therefore provide an additional layer of information that, together with amplitude and spectral cues, creates distinct vibroacoustic signatures that caterpillars can use to identify approaching intruders. Second, resident caterpillars frequently initiated defensive responses well before intruders reached their territories or made physical contact, sometimes responding within seconds, and when intruders were still several centimetres away. This rapid, distance-based discrimination is difficult to explain through visual or chemical cues alone, given caterpillars' limited visual capacity (England et al., 2026) and the temporal constraints associated with chemical cue transmission. Third, caterpillars suspended on silk threads after jumping overwhelmingly remained hanging until predators departed or became motionless, strongly suggesting that hanging larvae monitored leaf vibrations via their silk lifelines to assess when it was safe to return. While we did not directly measure vibrations transmitted through silk lifelines in our study, substrate-borne vibrations represent the most parsimonious sensory cue available to hanging caterpillars for assessing predator presence and activity on the leaf.

Our results provide several lines of evidence that vibrations play a key role in discrimination. However, an important caveat is that our experiments did not isolate substrate-borne vibrations from other potential sensory cues that may have been simultaneously available, such as visual cues or air currents generated by moving intruders, or chemical cues released by predators. To further test the role of vibrations, future experiments could employ playback methods (e.g. Castellanos and Barbosa, 2006) or vibration-isolation methods such as a ‘cut-leaf’ experiment (e.g. Guedes et al., 2012). Taken together, our findings along with previous research (Matheson et al., 2025), despite potential experimental constraints, add to a growing body of evidence that these tiny caterpillars live within a rich vibratory landscape, where substrate-borne vibrations play a central role in both intruder discrimination and social communication (Matheson et al., 2025).

This begs the question: how do these tiny larvae detect and discriminate between different vibrations? There is increasing evidence that caterpillars and other holometabolous larvae use vibrations to detect and discriminate between enemies and engage in complex social interactions (Yack, 2016; Yack and Yadav, 2022). Currently, vibration-sensitive sensory organs have not yet been definitively identified in larval insects, although candidate structures include trichoid sensilla and scolopidia in the prolegs (Yack and Yadav, 2022). The discovery of such sensors will fulfil an important knowledge gap in insect sensory biology.

### Conclusion

This study demonstrates that substrate-borne vibrations play multifaceted roles in the predator–prey interactions of neonate warty birch caterpillars. While vibratory signals evolved primarily for territorial communication rather than antipredator defence, vibrations constitute critical information sources for predator detection and discrimination. Resident caterpillars employ sophisticated vibration-mediated threat assessment, rapidly identifying intruder types based on distinctive vibratory signatures and deploying context-appropriate defensive strategies from a flexible behavioural repertoire. This demonstrates remarkable perceptual sophistication in tiny first-instar larvae. These findings expand our understanding of predator–prey interactions in the vibroscape (Virant-Doberlet et al., 2019, 2023).

## Supplementary Material

10.1242/jexbio.252329_sup1 Supplementary information
