# Supplementary material for: Tiny caterpillars assess threats by the footsteps of their enemies
Source: J Exp Biol. 2026 Jun 4;229(11):jeb252329. doi: 10.1242/jeb.252329 (PMC13288082; doi:10.1242/jeb.252329)
Supplement: Supplementary information [file jexbio-229-252329-s1.pdf]

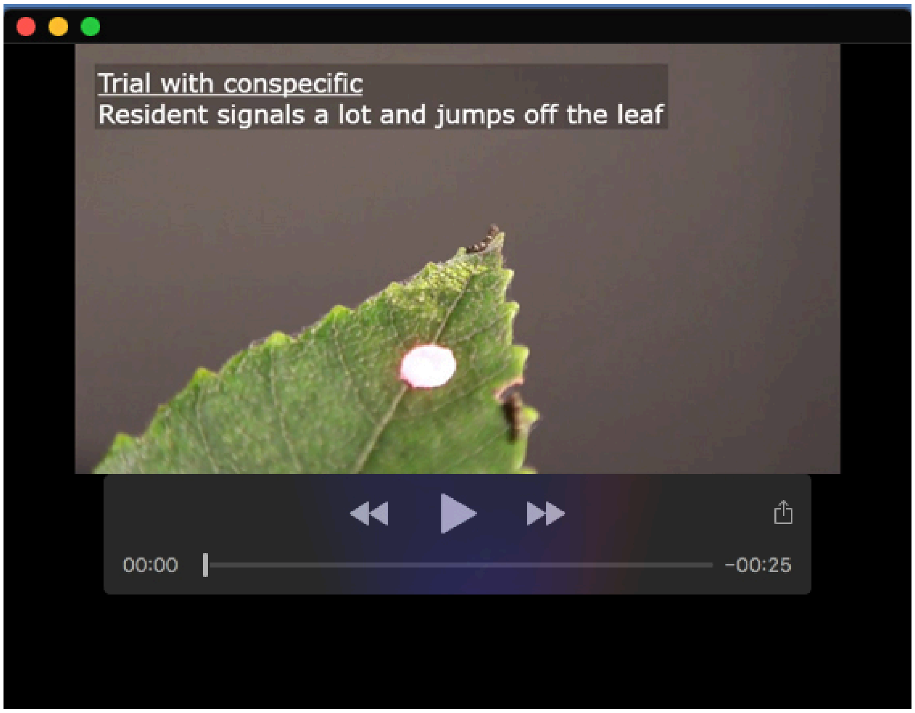

Movie 1. A resident first instar *F. bilineata* larva on its territory is approached by a conspecific larva, signals, and then jumps off the leaf on a silk lifeline when the intruder enters the territory and makes contact.

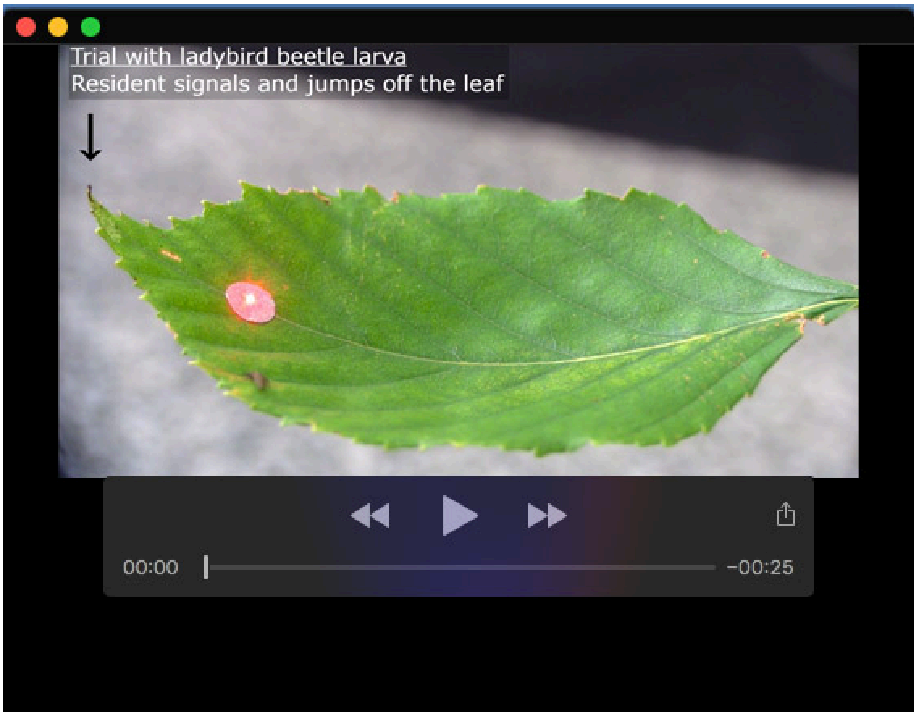

Movie 2. A resident first instar *F. bilineata* larva on its territory is approached by a predatory ladybird beetle larva. The resident larva signals briefly and then becomes immobile before it jumps off the leaf. The resident returns to the leaf after the predator, and respective vibrations, are gone. A black arrow indicates the resident larva's position.

Journal of Experimental Biology • Supplementary information

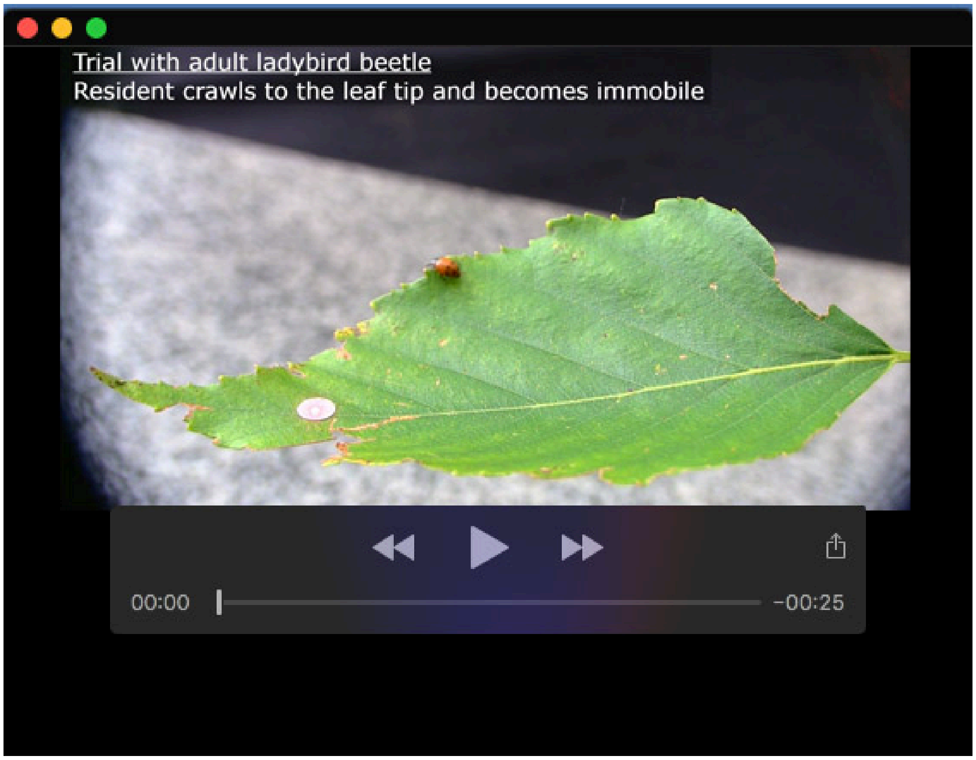

Movie 3. Three clips showing behavioural responses of a resident first instar *F. bilineata* larva on its territory to an approaching predatory adult ladybird beetle. In the first clip, the resident larva moves toward the leaf tip and then becomes immobile when the ladybird beetle arrives on its territory. In the second clip, the resident larva jumps off the leaf as the ladybird beetle approaches, then returns to the leaf after the predator, and respective vibrations, are gone. In the third clip, a separate resident larva remains immobile and is subsequently attacked and consumed by the ladybird beetle. A black arrow indicates the resident larva's position in each clip.
